# Lane Detection Aided Online Dead Reckoning for GNSS Denied Environments

**DOI:** 10.3390/s21206805

**Published:** 2021-10-13

**Authors:** Jinhwan Jeon, Yoonjin Hwang, Yongseop Jeong, Sangdon Park, In So Kweon, Seibum B. Choi

**Affiliations:** 1 Department of Mechanical Engineering, Korea Advanced Institute of Science and Technology, Daejeon 34141, Korea; jordan98@kaist.ac.kr (J.J.); yoonjinh@kaist.ac.kr (Y.H.); 2 The Robotics Program, Korea Advanced Institute of Science and Technology, Daejeon 34141, Korea; yongseop@kaist.ac.kr; 3 School of Electrical Engineering, Korea Advanced Institute of Science and Technology, Daejeon 34141, Korea; sangdon.park@kaist.ac.kr (S.P.); iskweon77@kaist.ac.kr (I.S.K.)

**Keywords:** dead reckoning, lane detection, sensor fusion, multimodal system

## Abstract

With the emerging interest of autonomous vehicles (AV), the performance and reliability of the land vehicle navigation are also becoming important. Generally, the navigation system for passenger car has been heavily relied on the existing Global Navigation Satellite System (GNSS) in recent decades. However, there are many cases in real world driving where the satellite signals are challenged; for example, urban streets with buildings, tunnels, or even underpasses. In this paper, we propose a novel method for simultaneous vehicle dead reckoning, based on the lane detection model in GNSS-denied situations. The proposed method fuses the Inertial Navigation System (INS) with learning-based lane detection model to estimate the global position of vehicle, and effectively bounds the error drift compared to standalone INS. The integration of INS and lane model is accomplished by UKF to minimize linearization errors and computing time. The proposed method is evaluated through the real-vehicle experiments on highway driving, and the comparative discussions for other dead-reckoning algorithms with the same system configuration are presented.

## 1. Introduction

Precise positioning and localization techniques for modern land vehicles have been widely implemented for the purpose of advanced driving assist system and autonomous driving capability. Global Navigation Satellite System (GNSS) has been adopted as a primary option to obtain the position and velocity of the vehicle. Since land vehicles are designed to be driven on the road, the positioning accuracy of GNSS can be compensated with the road map from Geographic Information System (GIS) [1,2,3,4] for the conventional navigation purpose, and even with the real time kinematics (RTK) techniques [5,6], its positioning performance can be improved up to centimeter-level accuracy.

Despite the outstanding accuracy and wide coverage of RTK GNSS, the satellite signal outage and multipath error in GNSS-denied area, such as densely built city, underpass, or indoor area, significantly threaten the reliability of the GNSS measurement [7,8]. To overcome the environmental limitation of the GNSS measurement, several alternative navigation methods with other types of measurements are introduced to ensure the consistency of positional information and improve the minimum performance under a poor satellite signal condition [9,10,11]. Those methods, well known as dead-reckoning (DR), are based on the cumulative process of relative change in the speed and direction from the latest known position.

Inertial Navigation System (INS) has been commonly adopted to complement GNSS [12,13,14,15,16]. During the period that GNSS signal is unavailable, INS estimate the position, velocity and attitude by integrating the inertial measurements, such as acceleration and angular rate. With the advancement of computing technology, visual sensors have been used as positioning devices [17,18,19,20,21,22,23,24]. Modern silicons allow the real-time processing of high-resolution stereo images, which can directly compute the motion of camera set, and uses machine learning to estimate 3-axis motion from a monocular system. Recently, lidar-based localization methods are also introduced to perform precise positioning with point cloud maps in sub-meter accuracy.

However, considering the fact that GNSS is still considered as a primary device for navigation systems, it is obvious that those alternative positioning methods have their own limitations. INS have been widely used in various fields, including military and aerospace technologies, where the performance and reliability are top priorities. Although the nature of INS convinces near-perfect motion estimation theoretically, there occurs an inevitable error in reality without external aiding due to the imperfection of sensor measurements. Visual odometry [25,26] and SLAM [27,28,29] estimate the ego-motion of the sensor, by comparing the positional changes of surrounding environments and reducing error accumulation using the historical measurements. The main drawback of methods based on external sensing is the result easily affected by the condition of surrounding environment. When the surrounding environment is not suitable to perform feature extraction and matching, for instance, foggy or rainy weather, low intensity, or highly homogeneous scenes, DR based on environmental sensing easily fails.

On the other hand, applying those advanced positioning and localization techniques on mass-production vehicles are considered premature for several reasons. Currently, the mainstream environmental sensing equipment for consumer cars consists of monocular vision for lane detection, frontal radar for collision avoidance, and GNSS for navigation system. It is known that monocular vision system has scale ambiguity, which disturbs absolute motion estimation, and radar has highly sparse feature points that can be easily lost. Moreover, global positioning methods based on map-matching approaches require a large amount of digital map data, and there still remain numerous works to implying the high-definition map (HD map)-based localization in public.

**Figure 1:**
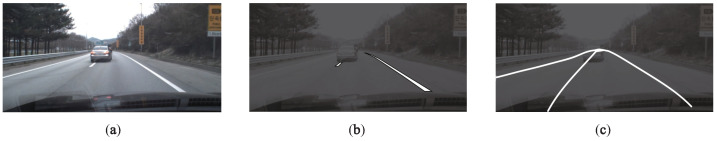
Example for lane detection output difference according to approaches. (**a**) Original Image, (**b**) Feature-based Lane Detection, (**c**) Learning-based Lane Detection.

In order to mitigate the shortcomings of DR performance of monocular vision and inertial measurement, this research focused on lane detection results from camera. Unlike feature extraction, learning-based lane detection gives a highly consistent result from same images. Recently, as a remarkable evolution in neural-network and artificial intelligence, learning-based lane detection models [30,31,32] shows better robustness than conventional machine vision approaches in challenging situations, such as varying shadows and image occlusions by moving objects. Figure 1 presents the lane detection results from both feature-based and learning-based approaches. For real driving scenes like highway driving, those challenges happen everyday, and therefore, learning-based lane detection is widely adopted in production vehicles.

In this paper, we propose a DR method that uses robust lane detection results from the learning-based lane detection model [32]. As explained above, using standalone INS will gradually lead to drifting issues for vehicle kinematic/dynamic state variables, e.g., vehicle roll angle, bank angle of road surface and vehicle heading angle. By using the robust lane detection results, these drifting problems are to be compensated and therefore will be regulated to much smaller magnitudes compared to standalone INS. Moreover, using lane detection results for correction show higher performance and better computational cost than the state-of-the-art vision-based methods in real-world experiments.

We summarize the main contributions of our work as below:

- We proposed a novel filter design that combines learning-based lane detection results with IMU mechanization for accurate vehicle localization in GNSS denied environments.
- Accurate online vehicle localization was achieved for various road geometry and environment conditions, verifying the robustness of our proposed method.

The rest of the paper is organized as follows. In Section 2, vehicle kinematics model and observer model are introduced. In Section 3, filter selection and implementation process are illustrated. In Section 4, experiment scenarios, vehicle set up and various dataset from the experiment are explained. In Section 5, the result of lane detection-aided DR is presented and is compared with other visual odometry-based localization algorithms. Finally, in Section 6, the conclusion of this research will be illustrated.

## 2. System Modeling

**Figure 2:**
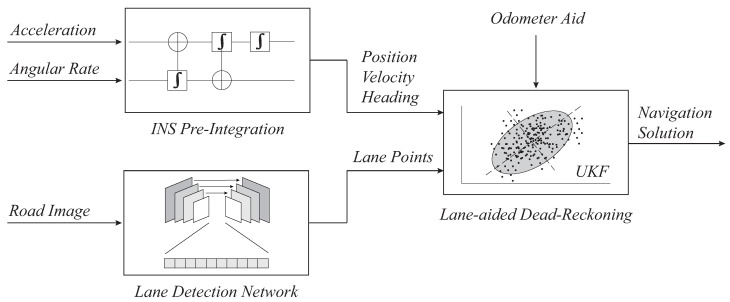
Overall architecture of land-aided dead-reckoning system.

In this section, vehicle kinematics and observer model design process will be explained thoroughly. To design a kinematic model that operates inside the filter, we first need to consider the overall framework of our research. From Figure 2, we can see that, using IMU measurement and lane detection results, the system should output reliable vehicle localization data.

**Figure 3:**
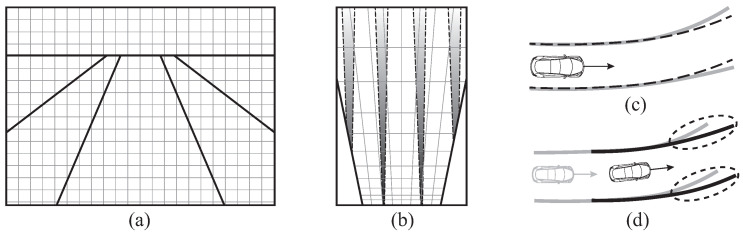
Potential error sources when using lane detection for vehicle localization: (**a**) Original road image in perspective view; (**b**) Blurred lane estimation accuracy along preview distance in global frame; (**c**) Effects of vehicle attitude and road inclination in lane detection result; (**d**) Mismatched lane lines in successive frames.

As shown in Figure 3, the result from vision-based lane detection might be degraded for various reasons, such as motion of vehicle, luminous intensity or shape and color of lane lines. In the purpose of rejecting outliers in the lane detection results and securing the consistent performance of position estimation, a vehicle kinematics-based observer model will be implemented based on this general framework.

### 2.1. Vehicle Kinematics Model

Vehicle kinematics follow the process of INS mechanization and a total of 9 vehicle states are propagated. Vehicle states and inputs are shown below.

(1) $$X_{k-1}={\left[\begin{array}{ccccccccc} x_{k-1} & y_{k-1} & z_{k-1} & v_{k-1}^{x} & v_{k-1}^{y} & v_{k-1}^{z} & \phi_{k-1} & \theta_{k-1} & \psi_{k-1} \end{array}\right]}^{T}$$

(2) $$u_{k-1}={\left[\begin{array}{cccccc} a_{k-1}^{x} & a_{k-1}^{y} & a_{k-1}^{z} & \omega_{k-1}^{x} & \omega_{k-1}^{y} & \omega_{k-1}^{z} \end{array}\right]}^{T}$$

ϕ,θ,ψ represent the Euler angles of the vehicle frame. At the initial step, we initialize all the states and vehicle attitude matrix according to the IMU measurements. Suppose that the vehicle attitude matrix at timestep (k−1) is ${\left(C_{b}^{n}\right)}_{k-1}$, skew matrix computed from Euler angles is $S_{k-1}$ and norm of $\omega_{k-1}^{3\times 1}T$ as $\left|\right|\omega_{k-1}^{3\times 1}T\left|\right|$, then we can first update the vehicle attitude matrix using the angular velocity input and compute the vehicle acceleration in the navigation frame.

(3) $$\begin{array}{cc} \; & \left[\begin{array}{c} {\left(a_{k-1}^{x}\right)}_{n}\\ {\left(a_{k-1}^{y}\right)}_{n}\\ {\left(a_{k-1}^{z}\right)}_{n} \end{array}\right]={\left(C_{b}^{n}\right)}_{k-1}\left[\begin{array}{c} a_{k-1}^{x}\\ a_{k-1}^{y}\\ a_{k-1}^{z} \end{array}\right]-\left[\begin{array}{c} 0\\ 0\\ 9.8 \end{array}\right] \end{array}$$

(4) $$\begin{array}{cc} \; & S_{k-1}=skew\left(\left[\begin{array}{ccc} \omega_{k-1}^{x}T & \omega_{k-1}^{y}T & \omega_{k-1}^{z}T \end{array}\right]\right)=skew\left({\left(\omega_{k-1}^{3\times 1}\right)}^{T}\;T\right) \end{array}$$

(5) $$\begin{array}{cc} \; & {\left(C_{b}^{n}\right)}_{k}={\left(C_{b}^{n}\right)}_{k-1}+I_{3\times 3}+\left(\frac{\sin\left|\right|\omega_{k-1}^{3\times 1}T\left|\right|}{\left|\right|\omega_{k-1}^{3\times 1}T\left|\right|}\right)S_{k-1}+\left(\frac{1-\cos\left|\right|\omega_{k-1}^{3\times 1}T\left|\right|}{\left|\right|\omega_{k-1}^{3\times 1}T{\left|\right|}^{2}}\right)S_{k-1}^{2} \end{array}$$

*T* is the timestep interval and is 0.05 s (20 Hz) during the simulation process. Using the updated vehicle attitude matrix and acceleration data, we can propagate the updated Euler angles, velocity vector and position vector.

(6) $$\begin{array}{cc} \; & x_{k}=x_{k-1}+v_{k-1}^{x}T+\frac{1}{2}{\left(a_{k-1}^{x}\right)}_{n}\;T^{2} \end{array}$$

(7) $$\begin{array}{cc} \; & y_{k}=y_{k-1}+v_{k-1}^{y}T+\frac{1}{2}{\left(a_{k-1}^{y}\right)}_{n}\;T^{2} \end{array}$$

(8) $$\begin{array}{cc} \; & z_{k}=z_{k-1}+v_{k-1}^{z}T+\frac{1}{2}{\left(a_{k-1}^{z}\right)}_{n}\;T^{2} \end{array}$$

(9) $$\begin{array}{cc} \; & v_{k}^{x}=v_{k-1}^{x}+{\left(a_{k-1}^{x}\right)}_{n}\;T \end{array}$$

(10) $$\begin{array}{cc} \; & v_{k}^{y}=v_{k-1}^{y}+{\left(a_{k-1}^{y}\right)}_{n}\;T \end{array}$$

(11) $$\begin{array}{cc} \; & v_{k}^{z}=v_{k-1}^{z}+{\left(a_{k-1}^{z}\right)}_{n}\;T \end{array}$$

(12) $$\begin{array}{cc} \; & \phi_{k}=atan2\left(\;{\left(C_{b}^{n}\right)}_{k}\left(2,2\right)\;,\;{\left(C_{b}^{n}\right)}_{k}\left(3,3\right)\;\right) \end{array}$$

(13) $$\begin{array}{cc} \; & \theta_{k}=-\arcsin\left(\;{\left(C_{b}^{n}\right)}_{k}\left(1,3\right)\;\right) \end{array}$$

(14) $$\begin{array}{cc} \; & \psi_{k}=atan2\left(\;{\left(C_{b}^{n}\right)}_{k}\left(1,2\right)\;,\;{\left(C_{b}^{n}\right)}_{k}\left(1,1\right)\;\right) \end{array}$$

Arranging the results above, propagated vehicle states can be written as following.

(15) $$\begin{array}{cc} X_{k} & ={\left[\begin{array}{ccccccccc} x_{k} & y_{k} & z_{k} & v_{k}^{x} & v_{k}^{y} & v_{k}^{z} & \phi_{k} & \theta_{k} & \psi_{k} \end{array}\right]}^{T}\\ \; & =f(X_{k-1},u_{k-1}) \end{array}$$

### 2.2. Observer Model

**Figure 4:**
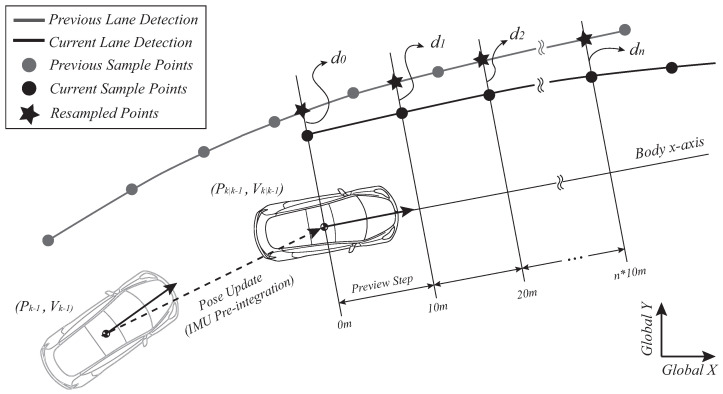
Observer Model: Predicting lateral distance to the previewed lane.

In order to update the vehicle states by using lane detection results, we can first think of using the previous step lane geometry, as shown in Figure 4.

Considering filter implementation at Section 3, previous step lane detection results and previous step vehicle position estimates are used to create the previewed lane geometry (previous sample points) at the ${\left(k-1\right)}^{\mathrm{th}}$ step. After the IMU pre-integration introduced at Section 2.1, we can resample points on the previous lane geometry by linear interpolation. This can be compared with the actual measurement made at the $k^{\mathrm{th}}$ step (current sample points) for vehicle position error compensation.

The actual implementation starts off with creating the lane geometry information with ${\left(k-1\right)}^{\mathrm{th}}$ step updated vehicle position and ${\left(k-1\right)}^{\mathrm{th}}$ step lane detection results. Suppose that we are obtaining the global coordinates for $n^{\mathrm{th}}$ previewed left lane point $\left({\left(x_{n}^{l}\right)}_{k-1},\;{\left(y_{n}^{l}\right)}_{k-1}\right)$. The coordinates can be computed as below.

(16) $$\begin{array}{cc} \; & {\left(x_{n}^{l}\right)}_{k-1}=x_{k-1}+10n\;\cos\left(\psi_{k-1}\right)-{\left(l_{n}\right)}_{k-1}\;\sin\left(\psi_{k-1}\right) \end{array}$$

(17) $$\begin{array}{cc} \; & {\left(y_{n}^{l}\right)}_{k-1}=y_{k-1}+10n\;\sin\left(\psi_{k-1}\right)+{\left(l_{n}\right)}_{k-1}\;\cos\left(\psi_{k-1}\right) \end{array}$$

${\left(l_{n}\right)}_{k-1}$ is the lateral distance to the 10nm (longitudinal) previewed left lane point measured by the lane detection model. These coordinates for all the previewed points are the previous sample points in Figure 4. Then, we convert previous sample points coordinates from global frame to $k^{\mathrm{th}}$ vehicle body frame (IMU pre-integrated). Frame transformation of $n^{\mathrm{th}}$ previewed left lane point can be done as follows.

(18) $$\begin{array}{cc} \; & {\left(\psi_{n}^{rel}\right)}_{k}=\psi_{k}-atan2\left(\;{\left(y_{n}^{l}\right)}_{k-1}-y_{k},\;{\left(x_{n}^{l}\right)}_{k-1}-x_{k}\right) \end{array}$$

(19) $$\begin{array}{cc} \; & {\left(L_{n}^{l}\right)}_{k}=\sqrt{{\left({\left(x_{n}^{l}\right)}_{k-1}-x_{k}\right)}^{2}+{\left({\left(y_{n}^{l}\right)}_{k-1}-y_{k}\right)}^{2}} \end{array}$$

(20) $$\begin{array}{cc} \; & {\left(x_{n}^{l}\right)}_{k-1}^{b}={\left(L_{n}^{l}\right)}_{k}\cos{\left(\psi_{n}^{rel}\right)}_{k} \end{array}$$

(21) $$\begin{array}{cc} \; & {\left(y_{n}^{l}\right)}_{k-1}^{b}={\left(L_{n}^{l}\right)}_{k}\sin{\left(\psi_{n}^{rel}\right)}_{k} \end{array}$$

${\left(\psi_{n}^{rel}\right)}_{k}$ in Equation (18) represents the relative angle of the previewed lane point measured from the vehicle body *x* axis. ${\left(L_{n}^{l}\right)}_{k}$ in Equation (19) is the 2D Euclidean distance from the IMU pre-integrated vehicle position and the $n^{\mathrm{th}}$ left lane point. The superscript *b* at Equations (20) and (21) mean that they are measured from the vehicle body frame. Note that the subscript of ${\left(x_{n}^{l}\right)}_{k-1}^{b}$ in Equation (20) is (k−1) because we are simply transforming ${\left(x_{n}^{l}\right)}_{k-1}$, which is the x coordinate of the previous sample point.

For measurement update, we can compare ${\left(y_{n}^{l}\right)}_{k-1}^{b}$ with ${\left(l_{n}\right)}_{k}$, which is the $k^{\mathrm{th}}$ step lane detection result of $n^{\mathrm{th}}$ previewed left lane point. IMU pre-integration process error can be compensated through this step. Other than the lane information, we also use vehicle longitudinal velocity for the measurement model.

(22) $$v_{k}^{b}=v_{k}^{x}\cos\left(\psi_{k}\right)+v_{k}^{y}\sin\left(\psi_{k}\right)$$

Combining the lateral distances of previewed points(*n* points for left and right lanes) and vehicle longitudinal velocity, the measurement prediction matrix can be written as follows.

(23) $$\begin{array}{cc} Z_{k} & ={\left[\begin{array}{cccccc} v_{k}^{b} & {\left(y_{1}^{l}\right)}_{k-1}^{b} & {\left(y_{1}^{r}\right)}_{k-1}^{b} & \cdots & {\left(y_{n}^{l}\right)}_{k-1}^{b} & {\left(y_{n}^{r}\right)}_{k-1}^{b} \end{array}\right]}^{T}\\ \; & =h(X_{k},u_{k-1}) \end{array}$$

Having *n* preview points for each lane, the size of the measurement prediction matrix will be $R^{(2n+1)\times 1}$. For measurement update, we organize the actual measurement matrix as below.

(24) $$Y_{k}={\left[\begin{array}{cccccc} {\left(v_{k}^{b}\right)}^{m} & {\left(l_{1}\right)}_{k} & {\left(r_{1}\right)}_{k} & \cdots & {\left(l_{n}\right)}_{k} & {\left(r_{n}\right)}_{k} \end{array}\right]}^{T}$$

${\left(v_{k}^{b}\right)}^{m}$ represents the longitudinal velocity actually measured by IMU. Using $Z_{k},\;Y_{k}$, we can update the vehicle states at the measurement update step, introduced at the next section.

## 3. Filter Design

### 3.1. Filter Selection and Framework

Nearly every vehicle localization problem is approached by using a filter that fits the proposed prediction/observation model and available data type well. The most popular filters are extended Kalman filter(EKF), unscented Kalman filter(UKF), and particle filter(PF) which show reliable performance for nonlinear or complex models.

Extended Kalman filter solves the nonlinear estimation problem by linearizing state and/or measurement equations and applying the standard Kalman filter formulas to the resulting linear estimation problem. The linearization yields to approximation errors, which the filter does not take into account in the prediction/update steps. Therefore, EKF error estimates tend to underestimate state uncertainties. In comparison, UKF picks so-called sigma point samples from the filtering distribution and propagates/updates them through the nonlinear state and measurement models. The resulting weighted set of sigma points represents how the updated filtering distribution, which is then approximated as a moment matched Gaussian distribution. This state estimation results represent the state uncertainty better than the estimates obtained from the EKF with an increased computational cost. Similar to UKF, the PF method propagates particles, but the main difference is that the particles are selected in a probabilistic manner. Generally, PF shows higher time complexity than EKF and UKF, because a lot of particles are needed to represent the entire nonlinear model.

Since one of our goals in this research is to implement the real-time vehicle localization method, we can see that PF is not an appropriate candidate for filter design. Taking our system into consideration, for GNSS denied situations with no precise map available, the only applicable measurement for update step is lane detection result. However, the output of lane detection model has high uncertainty for far preview distances, which may lead to huge error accumulation for EKF update process. Cancelling out the candidates, we finally have UKF as our filter structure.

**Figure 5:**
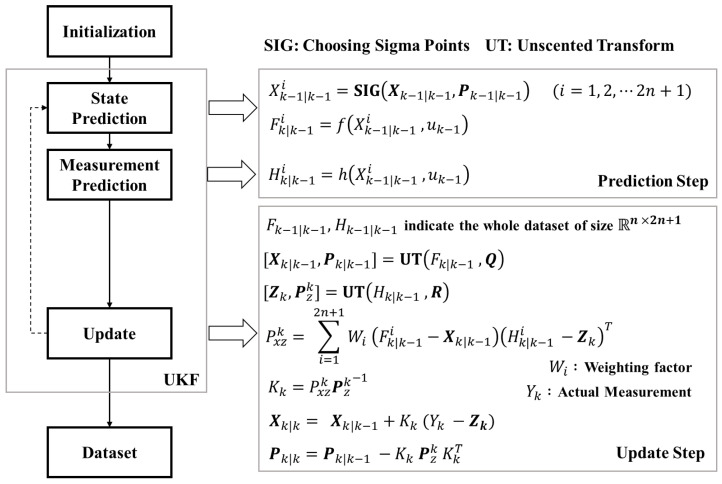
Simplified framework of UKF method.

From the subsections below, simple implementation of the UKF will be illustrated in the same order as the flowchart shown in Figure 5. Note that the variables used in this section are slightly modified from the ones at Section 2, adopting the Kalman filter notation.

### 3.2. Prediction Step

Before entering the main filtering loop, the initialization of all the state variables is done by using the GNSS/INS and vision data. Assuming that at least the initial conditions are very accurate, the variance values of all the states inside the covariance matrix were initially set as low quantities. Using the state variable format from Section 2, we can rewrite the state propagation equation in the KF notation,

(25) $$\begin{array}{cc} u_{k-1} & ={\left[\begin{array}{cccccc} a_{k-1}^{x} & a_{k-1}^{y} & a_{k-1}^{z} & \omega_{k-1}^{x} & \omega_{k-1}^{y} & \omega_{k-1}^{z} \end{array}\right]}^{T} \end{array}$$

(26) $$\begin{array}{cc} X_{k|k-1} & =f(X_{k-1|k-1},u_{k-1}) \end{array}$$

where function f is the state propagation function introduced at Section 2.

Then, the measurement prediction step can also be rewritten as follows.

(27) $$Z_{k}=h\left(X_{k|k-1},\;u_{k-1}\right)$$

For the simplicity of an explanation, extracting sigma points and performing unscented transform were not mentioned in the Equations (26) and (27). Furthermore, the prediction step for state covariance matrix was skipped. Detailed information about the implementation process is shown in Figure 5.

### 3.3. Update Step

At the update step, we have to compare the predicted measurement with the actual measurements. Referring to the observer design at Section 2.2, state update can also be described in the KF form.

(28) $$X_{k|k}=X_{k|k-1}+K_{k}\left(Y_{k}-Z_{k}\right)$$

The remaining filter implementation is done according to the flowchart of Figure 5. As the simulation loop continues, $X_{k|k}$ and $P_{k|k}$ are saved for data analysis at Section 5.

## 4. Experiment

As mentioned in Section 1, our goal is to achieve accurate online vehicle localization for GNSS denied situations. Therefore, we have to compare the result of our proposed model with ground truth and other state-of-the-art visual odometry-based methods to prove the performance. The following sections describe the equipment used in the experiment, the geographical information of the test site, lane detection model, and its results in detail.

### 4.1. Experiment Setup and Scenarios

**Figure 6:**
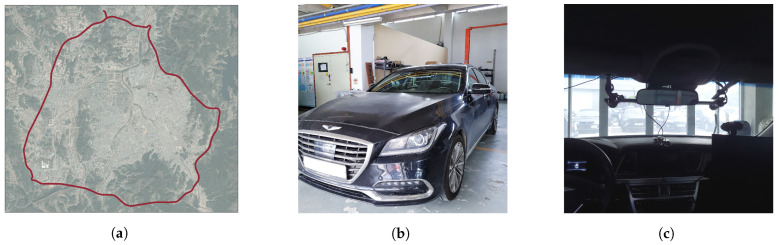
Test Environment and Experimental Setups are described in the figure. The experiment was done in Daejeon, South Korea, with a stereo camera-attached test vehicle (GENSIS G80 Sedan). (**a**) Experiment Trajectory, (**b**) GENESIS G80 Sedan (**c**) Stereo Camera.

In this research, we focus on the outdoor, especially highway (i.e., challenging feature extraction) situations because urban and indoor (e.g., parking lot) online vehicle localization can be achieved in high accuracy by existing visual odometry(VO) or SLAM methods. The experiment is carried out on the highway located in Daejeon, South Korea, and as shown in Figure 6a, the vehicle traveled approximately 52 km.

The test vehicle used for the research is GENESIS G80 Sedan, as shown in Figure 6b, and the camera used for forward view recording is the FLIR BLACKFLY model. Two monocular cameras are attached to the vehicle in Figure 6c to perform as stereo camera. In order to compare the proposed methods with other VO methods, an industrial grade IMU, Xsens MTi-670g is also fastened to the stereo vision system, and calibrated with the vehicle body coordinate [33,34]. Finally, the CPU used for simulation is Intel Core i5-4690 CPU @ 3.50 GHz, and RAM of 16 GB.

For the performance evaluation of our proposed method in various situations, there is a need to slice the total vehicle trajectory into some specific scenarios. The scenarios are chosen mainly according to the lane geometry and the surrounding environments. The localization performance of our proposed method will be illustrated for each scenario at Section 5. At the beginning of each scenario, we assume that there is GNSS initialization. After the initialization, our proposed method and the other comparison methods are propagated without any GNSS update.

### 4.2. Lane Detection Model

In order to obtain lane fragments from collected images, a CRNN-based lane detection model, named “supercombo”, is adopted [35], which is currently implemented in commercial aftermarket ADAS systems. The model takes its input as two successive image frame and latest fully connected layer. The output of the model consists of four lane line candidates, two road boundary for left and right edge, lead vehicle position estimation, and path planning results. In this research, we use only two lane lines, for left and right lanes, since those two lane lines are also presented in other types of lane detection methods as the essential output. It is worth noting that the detected lane lines have their preview length up to 100 m, while the estimated accuracy decreases as preview length increases.

### 4.3. Lane Detection Results

**Figure 7:**
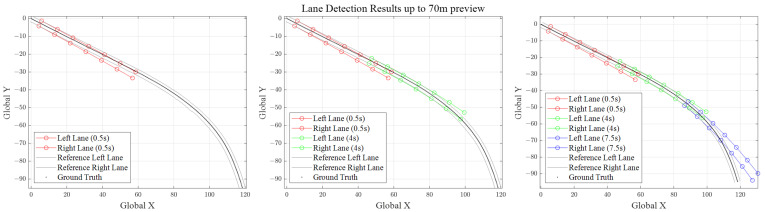
Lane Detection Results (0.5–7.5 s) with 70 m preview distance.

Before proceeding to DR implementation, we perform a pre-test of lane detection to validate the performance and reliability. Since the lane detection model is designed for a single-camera setup, the left camera from the stereo setup is used. The inference results of the lane detection model are presented in Figure 7, which describes the reprojected lane lines in the global coordinate. Ground truth of vehicle trajectory is obtained by OxTS RT3100, a commercial INS system for land vehicle test and survey.

**Figure 8:**
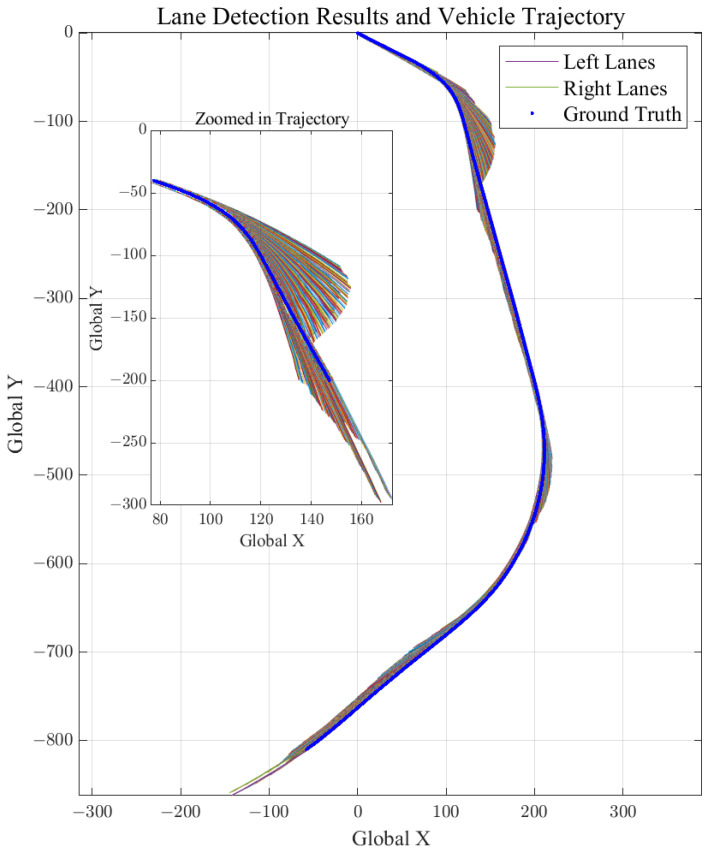
A lane detection result including up to 100 m previewed points is plotted with the vehicle position measured by OxTS RT3100 (vehicle position marked blue). As shown in the figure, longer preview distance show huge lateral deviation from the ground truth.

Figure 7 shows lane points for 3 different time steps with 70 m preview distance. Extending the preview distance up to 100 m and plotting for full simulation time of Scenario 1 (refer to Section 5.2), we can obtain Figure 8. Due to transformation error from image to real world coordinates and image distortion for far previewed distances, it is obvious that lateral distance data of 0 m previewed lane point is much more trustworthy compared to 100 m previewed lane point. As we can see in Figure 7 and Figure 8, further previewed lane points show huge deviations, especially at curvy road segments. However, this does not mean that the previewed lane point data should be discarded due to the high uncertainty. Although further previewed lane points have larger position errors, their existence implies curvature of the previewed lanes and restrains kinematic/dynamic vehicle states from diverging. This is a trade off problem, and will be discussed intensively at Section 5.6.1.

To sum up, the most accurate mapping possible from this dataset would be merging all the 0 m previewed lane points. Ground truth for this research can be thought of as 2 parts. First is the accurate vehicle position measured by RT and the second is 0m previewed lane points transformed into global fixed coordinates.

At Section 5, localization error will be computed by using the ground truth vehicle position obtained above. Other than the Euclidean distance error, heading angle difference will also be considered for analysis.

## 5. Results

### 5.1. Comparison Method: VO

**Figure 9:**
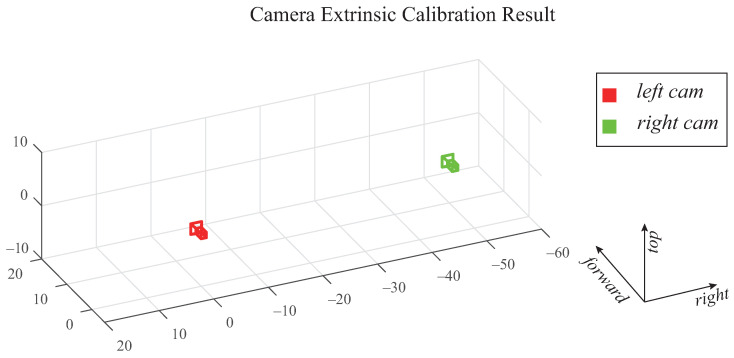
Extrinsic calibration result of stereo vision.

In order to evaluate the dead-reckoning performance of the proposed method, state-of-the-art visual odometry methods are also implemented. We chose VINS [36,37,38,39], top-ranked VO method in KITTI benchmarks, as competitive methods, since VINS have been designed for various types of system configurations, such as monocular vision, stereo vision, visual-inertial fusion, and even vehicle model fusion. It is worth noting that, for the fair comparison, the intrinsic and extrinsic parameters for cameras and IMU have been pre-calibrated with an open-sourced visual-inertial calibration library, kalibr [40]. Figure 9 shows the baseline of the stereo setup.

**Figure 10:**
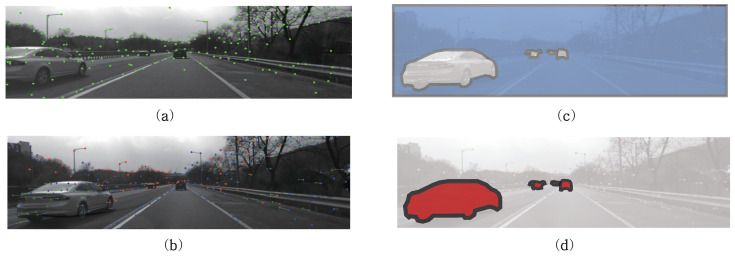
Disturbances on optical flow with moving traffics. (**a**) Matched Feature, (**b**) Optical Flow, (**c**) Static Scene, (**d**) Moving Scene.

However, unlike the indoor situation or urban driving scenes, the performance of VO is figured out to be degraded in the highway environment. Figure 10 shows the feature matching and calculated optical flow from a given image sequence. Since the background scene is nearly homogeneous, a large portion of features are extracted from surrounding vehicles. Moreover, the feature points on surrounding vehicles are relatively closer; hence, the effect of that points can be emphasized in the pose estimation result, while learning-based lane line detection shows consistent result, with or without surrounding vehicles.

**Figure 11:**
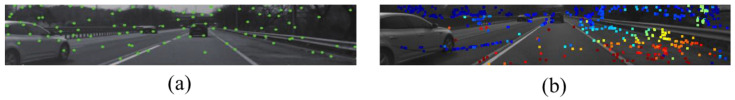
Point selection for VO in the homogeneous environment with moving traffic: (**a**) Selected points using feature matching. (**b**) Selected points using direct method.

In order to improve the performance degrading under the homogeneity of the scenery, the direct approach, specifically direct sparse odometry(DSO) [41], that uses the photometric error rather than the matching of selected set of feature points, has been adopted to competitive methods. The direct method shows more consistent ego-motion tracking performance. The sparse points from DSO also reflect the distinguishable characteristics in the middle of road surface, while the feature points from VINS tend to be biased on the corners on images as shown in Figure 11. However, under rapid changes in illuminance in the surrounding environment, such as direct sunlight toward camera or insufficient intensity in tunnels, the direct method shows the degraded performance, or fails occasionally.

Considering the drawbacks of comparison methods and to evaluate localization performance of our proposed method for specific lane geometry conditions, we extracted 4 scenarios from the highway drive. The result of localization for various scenarios will be presented in the following subsections, and overall analysis will be done at the end of the section. For simplicity, VINS Stereo + IMU is written as VINS1, VINS Stereo as VINS2 and VINS Mono + IMU as VINS3 for the RMSE comparison.

### 5.2. Scenario 1: Initial Stage

**Figure 12:**
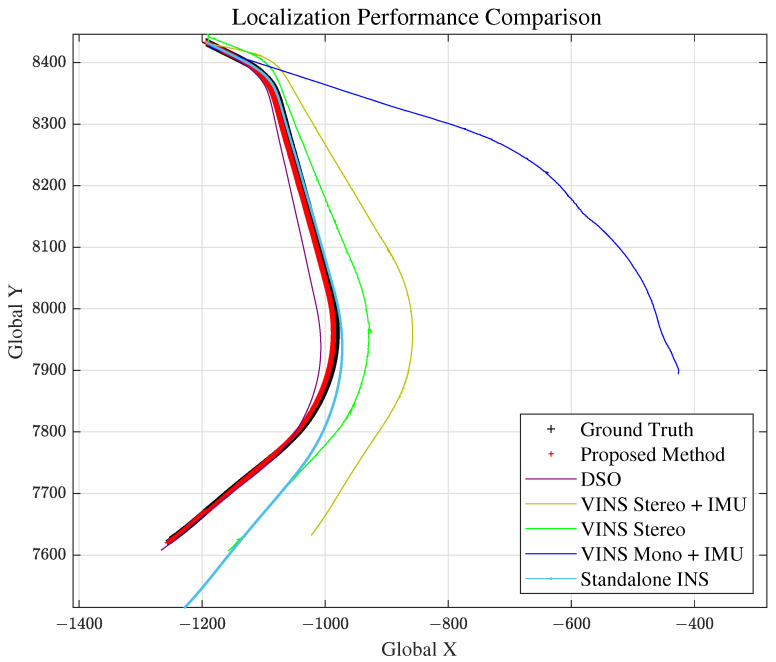
Scenario 1: Vehicle Localization with Various Methods.

**Figure 13:**
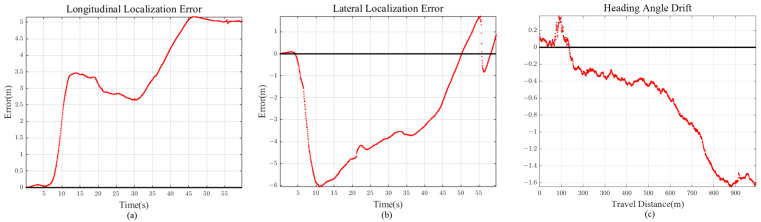
Scenario 1 (40 m Preview) (**a**) Longitudinal Error (**b**) Lateral Error (**c**) Heading Angle Drift.

The first scenario is the initial stage of the experiment, where a vehicle passes the tollbooth and enters the highway. This scene was chosen for evaluating standardized highway road geometry. As we can see from Figure 12, the ground truth lane does not have any extreme road geometry (high curvature, long straight path). The total travel distance and travel time of scenario 1 is approximately 992 m and 60 s, respectively. Localization comparison of methods is shown in Figure 12 and Figure 13 and Table 1.

### 5.3. Scenario 2: Straight Road

**Figure 14:**
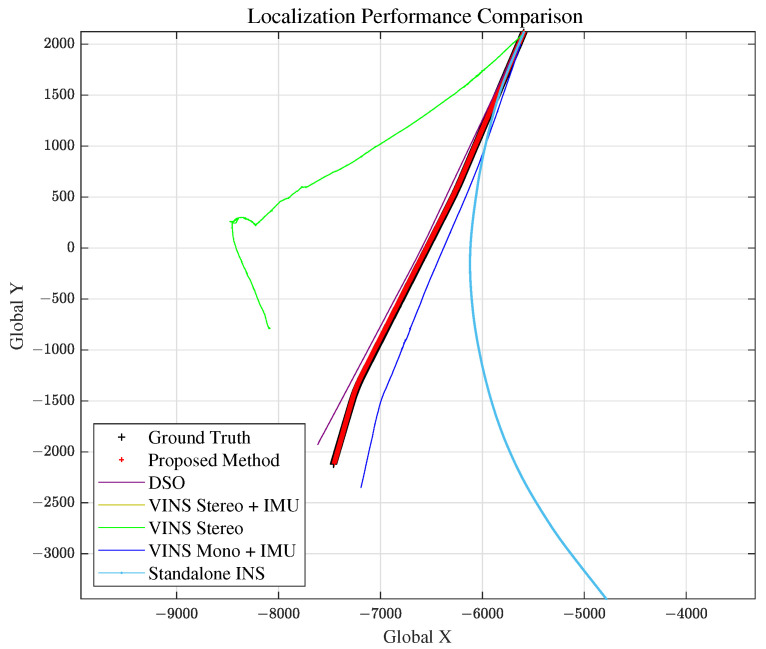
Scenario 2: Vehicle Localization with Various Methods.

**Figure 15:**
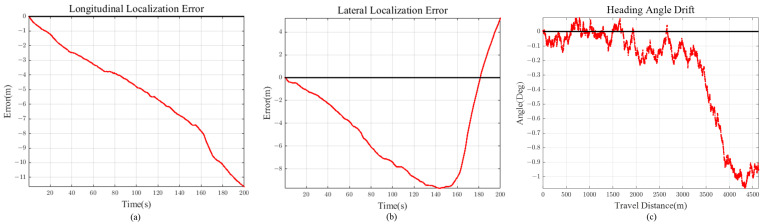
Scenario 2 (90 m Preview) (**a**) Longitudinal Error (**b**) Lateral Error (**c**) Heading Angle Drift.

Scenario 2 represents the case for a long straight road. This is to evaluate and analyze the longitudinal error magnitude for our proposed method. The total travel distance and travel time of scenario 2 are approximately 4.6 km and 200 s, respectively. Localization comparison of methods are shown in Figure 14 and Figure 15 and Table 2. VINS1(VINS Stereo + IMU) method failed in scenario 2.

### 5.4. Scenario 3: Curved Road

**Figure 16:**
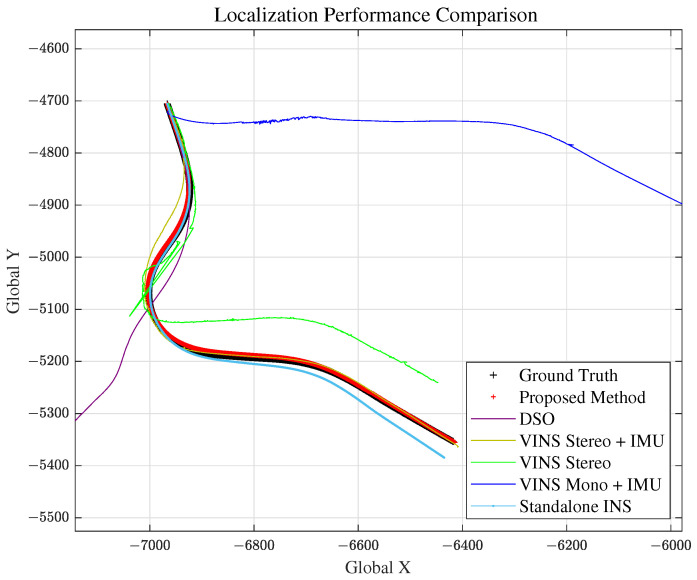
Scenario 3: Vehicle Localization with Various Methods.

**Figure 17:**
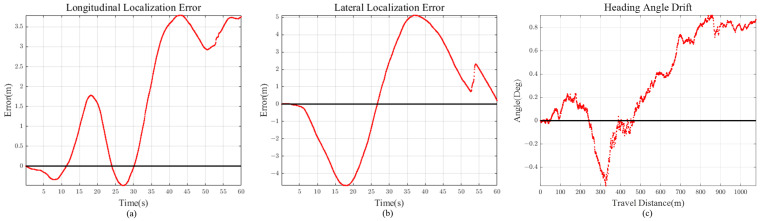
Scenario 3 (90 m Preview) (**a**) Longitudinal Error (**b**) Lateral Error (**c**) Heading Angle Drift.

Scenario 3 represents the case for curvy roads. High curvature trajectory was chosen from the ground truth data. The total travel distance and travel time of scenario 3 is approximately 1077 m and 60 s respectively. Localization results are shown in Figure 16 and Figure 17 and Table 3. Note that VINS1(VINS Stereo + IMU) localization result is close to the ground truth (marked yellow).

### 5.5. Scenario 4: Tunnels

**Figure 18:**
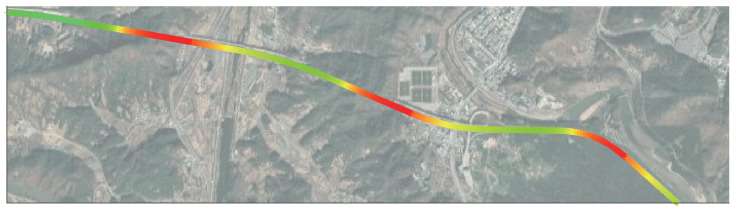
Scenario 4: GNSS signal outage in tunnels. Green denotes low dilution of precision, and red denotes high.

**Figure 19:**
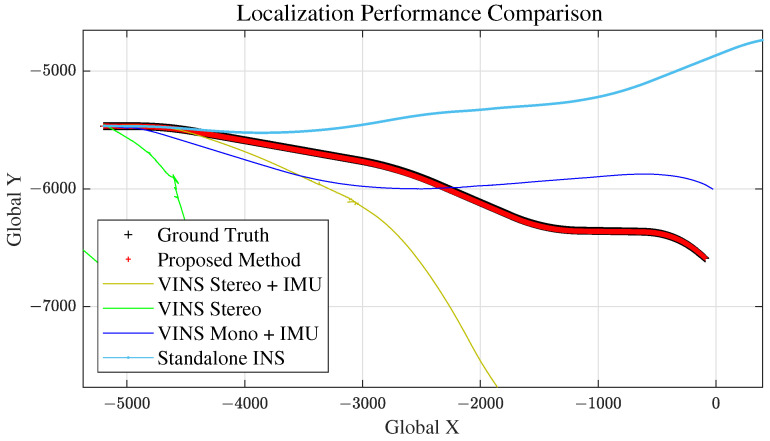
Scenario 4: Vehicle Localization with Various Methods.

**Figure 20:**
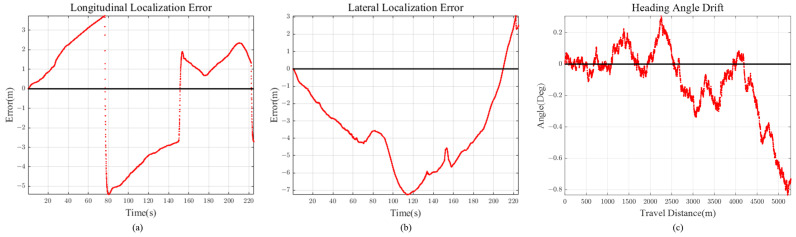
Scenario 4 (50 m Preview) (**a**) Longitudinal Error (**b**) Lateral Error (**c**) Heading Angle Drift.

As illustrated in Section 5.1, VO shows generally degraded performance at highway situations, and this is predicted to be more intensified in tunnels. In order to compare the localization performance of VO and proposed method for challenging feature extraction environments, scenario 4 was tested at Figure 18. Scenario 4 consists of 3 consecutive tunnels at the highway, as shown in Figure 18. The total travel distance and travel time of scenario 4 are approximately 5290 m and 225 s, respectively. Localization comparison of methods is shown in Figure 19 and Figure 20 and Table 4. In this scenario, DSO algorithm has failed.

### 5.6. Result Analysis

#### 5.6.1. Localization Performance for Varying Preview Distances

For 4 scenarios and their localization results from Table 1, Table 2, Table 3 and Table 4, we can observe that the localization performance of our proposed method is generally enhanced for further preview distances. As shown in Figure 8, although further previewed points have higher positional uncertainty, vehicle localization is stabilized by introducing forward lane geometry to the model update. Predicting the previewed point positions using the previous step lane detection measurements and vehicle position estimate “push” or “pull” the IMU mechanized vehicle position to the accurate location.

However, naively increasing the preview distance is not the optimal solution to accurate localization. Results from Table 1 and Table 4 show degrading localization performance after 40 m and 50 m preview distance, respectively. This is due to the inherent uncertainty of the lane detection results for far preview distances.

Therefore, we can conclude that optimal preview distances are different for various scenarios tested in this research, but localization performance is generally enhanced for longer preview distances.

#### 5.6.2. Longitudinal, Lateral Error and Heading Angle Drift of Proposed Method

It is intuitive that lane detection information helps vehicle localization in the lateral direction, but not for longitudinal direction. Observing the localization results for scenarios 1 to 4, we can see that our method shows accurate enough localization for both vehicle longitudinal and lateral directions. This is because previewed road curvature information “attracts” vehicle to the appropriate longitudinal position by measurement prediction model in Section 2.2, compensating for the accumulated longitudinal error.

If the road has high curvature, as shown in Section 5.4, longitudinal error is bounded with the help of previewed lane geometry. On the other hand, for scenario 2 (Figure 15), the error keeps on increasing because there is little feedback on the longitudinal direction for long straight road section (low road curvature). However, considering that the longitudinal error reached only 11 m after 4.6 km drive, this implies that even with small lane curvature feedback, longitudinal diverging tendency is maintained at a slow increasing rate.

Other than 2D Euclidean localization error, vehicle heading angle drift should also be considered for accuracy evaluation. For all the scenarios, we can see that the heading angle drift is regulated below 2 degrees of magnitude, even for long vehicle trajectories. Similar to the longitudinal error, heading angle is bounded by using the previewed lane geometry.

#### 5.6.3. Comparison with Other Methods

As we can see from Figure 12, Figure 14, Figure 16 and Figure 19 and RMSE comparison table for each scenario, our proposed method shows much better performance in estimating the vehicle position accurately, compared to other VO and standalone INS methods.

Except for scenario 4, at least 1 VO method showed adequate localization performance for each of the scenarios. However, in scenario 4, as mentioned in Section 5.5, the accuracy of VO methods is severely degraded. DSO failed, and VINS Stereo also totally diverged from the ground truth, and the same for the remaining 2 methods. This is due to the moving and homogeneous feature extraction in 3 consecutive tunnels. Our proposed method, however, uses the robust learning-based lane detection model, which means that “features” extracted for implementation(i.e., lane information) are consistent and very stable for analysis. Based on these lane detection results and proposed model, we succeeded in achieving accurate localization performance, even for the tunnel scenario.

## 6. Conclusions

### 6.1. Overall Summary

This study proposed a novel lane detection-based online dead-reckoning method in GNSS denied situations. Using IMU measurements and robust learning-based lane detection results as input to the system, vehicle kinematics and observer were designed. Vehicle position estimation was implemented by using unscented Kalman filter with the model structure at Section 2. For the various highway drive scenarios, the evaluation of localization performance of our proposed method was done by comparing with state-of-the-art VO methods and standalone INS results. Although positional shifting was inevitable for long trajectories, the proposed method showed much better results than the comparison sets by successfully restraining the diverging vehicle states with the previewed lane geometry. Moreover, it was verified that using previewed lane information up to certain distances enhanced the vehicle localization accuracy, but showed degrading performance when using too far-previewed lane detection results.

### 6.2. Future Research Direction

In this paper, we have implemented the vehicle localization method by fusing learning based lane detection results with IMU mechanization. However, this method does not take into account the pitching and rolling motion of the vehicle during the highway drive. Underestimation of these additional vehicle states may have caused unwanted localization errors in the proposed model and filter design. For further research, expansion of the vehicle and lane kinematics model to 3D scale, considering the rolling and pitching motion of vehicle, can be done to enhance localization accuracy.

Moreover, together with the loop closure algorithm, the proposed method could be further improved to create an accurate digital lane map along the vehicle trajectories, and is also expected to show enhanced performances when the lane lines are not presented continuously or rapidly changing.

## Figures and Tables

**Table 1:** Scenario 1 Localization Results (Trajectory Length: 992 m).

Dataset	10 m	20 m	30 m	40 m	50 m	60 m	70 m	80 m	90 m	INS	DSO	VINS1	VINS2	VINS3
RMSE (m)	14.56	15.79	8.09	5.06	7.34	9.32	9.31	9.75	9.83	41.11	48.66	132.4	55.89	456.0
RMSE Lat (m)	4.26	12.22	7.15	3.52	5.06	6.61	6.62	6.98	7.05	37.13	17.04	82.14	50.51	216.4
RMSE Long (m)	13.92	10.00	3.78	3.63	5.32	6.57	6.55	6.81	6.86	17.63	45.58	103.9	23.95	401.4
Max Error (m)	24.87	35.31	19.65	6.84	10.19	14.75	14.89	15.98	16.21	111.3	62.82	230.5	98.18	869.2

**Table 2:** Scenario 2 Localization Results (Trajectory Length: 4628 m) VINS1 Failed.

Dataset	10 m	20 m	30 m	40 m	50 m	60 m	70 m	80 m	90 m	INS	DSO	VINS1	VINS2	VINS3
RMSE (m)	447.9	161.6	62.26	22.24	12.56	9.05	8.89	8.60	8.56	1175	334.5	x	1315	342.4
RMSE Lat (m)	423.7	156.7	59.93	20.28	10.38	6.86	6.40	6.07	6.04	1161	93.63	x	1214	182.5
RMSE Long (m)	145.3	39.6	16.86	9.13	7.07	6.28	6.16	6.08	6.07	166.9	321.1	x	465.6	289.6
Max Error (m)	771.2	339.2	127.6	38.05	19.01	13.58	12.80	12.71	12.77	2984	490.7	x	1981	650.5

**Table 3:** Scenario 3 Localization Results (Trajectory Length: 1077 m).

Dataset	10 m	20 m	30 m	40 m	50 m	60 m	70 m	80 m	90 m	INS	DSO	VINS1	VINS2	VINS3
RMSE (m)	25.94	13.69	7.88	4.57	4.13	4.03	3.91	3.84	3.81	13.57	284.9	28.02	65.93	428.3
RMSE Lat (m)	23.80	13.08	6.00	2.57	2.68	3.08	3.01	3.05	3.05	12.85	130.9	11.33	31.81	379.4
RMSE Long (m)	10.32	4.03	5.09	3.78	3.15	2.61	2.49	2.33	2.28	4.36	253.0	25.62	57.75	198.6
Max Error (m)	67.87	37.57	19.32	7.95	6.46	6.45	6.24	6.12	6.07	35.5	723.2	61.04	196.5	632.5

**Table 4:** Scenario 4 Localization Results (Trajectory Length: 5290 m).

Dataset	10 m	20 m	30 m	40 m	50 m	60 m	70 m	80 m	90 m	INS	DSO	VINS1	VINS2	VINS3
RMSE (m)	753.5	152.1	46.21	10.92	5.12	5.26	5.43	5.61	5.66	990.6	x	1146	3914	1489
RMSE Lat (m)	695.0	146.9	44.67	10.32	4.31	4.44	4.63	4.85	4.92	950.8	x	1111	3647	313.0
RMSE Long (m)	291.2	39.32	11.84	3.57	2.78	2.82	2.83	2.82	2.80	277.9	x	280.8	1422	1422
Max Error (m)	1576	337.5	100.5	17.65	8.20	9.79	10.70	10.93	10.81	2088	x	2583	6946	2429

## Data Availability

The data presented in this study are available on request from the authors. The data are not publicly available due to privacy reasons.
